# Dynamic Distribution of Histone H4 Arginine 3 Methylation Marks in the Developing Murine Cortex

**DOI:** 10.1371/journal.pone.0013807

**Published:** 2010-11-03

**Authors:** Alexandra Chittka

**Affiliations:** Wolfson Institute for Biomedical Research, University College London, London, United Kingdom; Katholieke Universiteit Leuven, Belgium

## Abstract

**Background:**

Epigenetic modifications regulate key transitions in cell fate during development of the central nervous system (CNS). During cortical development the initial population of proliferative neuroepithelial precursor cells give rise to neurons and then glia in a strict temporal order. Neurogenesis and gliogenesis are accompanied by a switch from symmetric to asymmetric divisions of the neural precursor cells generating another precursor and a differentiated progeny. To investigate whether specific post-translational histone modifications define specific stages of neural precursor differentiation during cortical development I focussed on the appearance of two different types of histone arginine methylation, the dimethyl symmetric H4R3 (H4R3me2s) and dimethyl asymmetric H4R3 (H4R3me2a) in the developing mouse cortex.

**Methodology/Principal Findings:**

An immunohistochemical study of the developing cortex at different developmental stages was performed to detect the distribution of H4R3me2s and H4R3me2a modifications. I analysed the distribution of these modifications in: 1) undifferentiated neural precursors, 2) post-mitotic neurons and 3) developing oligodendrocyte precursors (OLPs) using lineage-specific and histone modification-specific antibodies to co-label the cells. I found that the proliferative neuroepithelium during the stage of mainly symmetric expansive divisions is characterised by the prevalence of H4R3me2s modification and almost no detectable H4R3me2a modification. However, at a later stage, when the cortical layers with post-mitotic neurons have begun forming, both H4R3me2a and H4R3me2s modifications are detected in the post-mitotic neurons and in the developing OLPs.

**Conclusions/Significance:**

I propose that the H4R3me2s modification forms part of the “histone code” of undifferentiated neural precursors. The later appearance of the H4R3me2a modifications specifies the onset of neurogenesis and gliogenesis and the commitment of the NSCs to differentiate. Thus, the sequential appearance of the two different H4R3 methylation marks may define a particular cellular state of the NSCs during their development and differentiation demonstrating the role of histone arginine methylation in cortical development.

## Introduction

Generation of a functional nervous system is a result of a highly specific developmental programme of events. During cortical development a founding population of neuroepithelial cells, the neural stem cells (NSCs), gives rise to all cellular types of the cortex: neurons, astrocytes and oligodendrocytes [1]. The generation of these cell types follows a strict temporal order, with neurogenesis preceding gliogenesis [2], [3], [4]. Importantly, with the progress of differentiation within the CNS, the differentiation potential of the NSCs becomes more restricted, providing the basis for the temporal regulation of differentiation within the developing cortex [1].

To a large extent the strict temporal order of differentiation observed during cortical development is regulated by epigenetic mechanisms which re-programme the genomes for lineage-specific “transcriptomes” by regulating chromatin structure [5]. The most prominent epigenetic modifications associated with developmental regulation of gene expression include methylation of DNA at the CpG dinucleotides by DNA methyltransferases and the post-translation modifications of histones [5], [6]. Histones can be acetylated on lysine or methylated on lysine or arginine residues [5], [7]. Such modifications modulate the compaction of chromatin and its general accessibility to transcriptional machinery. In general, lineage-specific transcription factors recruit the appropriate chromatin modifiers to induce changes in the chromatin accessibility and in this way orchestrate the appropriate “transcriptomes”. While an enormous effort has gone into uncovering the contributions of the lineage-specific transcription factors to the development of different cell lineages, much less is known about the contribution of specific global modifications which may specify particular cellular states as the NSCs transit through the temporal stages of their differentiation. Such modifications of histones provide a potential storage mechanism for heritable information which can be transmitted through mitotic divisions and subsequently “read” and interpreted by effector proteins. Specific inherited histone modifications and the reading effectors could induce the specific spatial and temporal gene expression by regulating accessibility of the DNA to transcriptional machinery during development and differentiation [8].

Previous work in murine oocytes during their maturation and pre-implantation development identified a series of stable and dynamic “epigenetic marks” associated with different developmental stages [6]. These included histone H3 lysine 9 methylation (H3K9me), H3 lysine 4 methylation (H3K4me) and histone H4/H2A serine 1 phosphorylation (H4/H2AS1ph) which were stable throughout the developmental stages investigated [6]. The dynamic and reversible ones included hyperacetylated histone H4 (H4ac), histone H3 arginine 17 methylation (H3R17me) and histone H4 arginine 3 methylation (H4R3me) [6].

To begin defining some of the global changes in post-translational histone modifications which accompany neural differentiation, I undertook an analysis of the distribution of two specific histone arginine modifications, histone H4 arginine 3 symmetric and asymmetric dimethylation. Arginine modifications are mediated by two classes of protein arginine methyltransferases (PRMTs), class I and II. Class I PRMTs place two methyl groups on the one nitrogen atom of the arginine guanidino group, generating an asymmetric dimethyl modification. Class II PRMTs, on the other hand, place two methyl groups on the two nitrogen atoms of the guanidino group generating symmetric dimethylation [9]. Interestingly, the two different types of histone arginine modifications, the symmetric and the asymmetric ones, tend to be associated more with transcriptional repression or activation, respectively, although this is not always the case [9]. There is also evidence that type I PRMTs which induce asymmetrical dimethylation of arginines are associated with cellular differentiation [10], [11]. However, the contribution of type II class of PRMTs is less clear, but there are reports which suggest that symmetrical dimethylation of arginines is associated with the less differentiated cellular state [12], [13].

To test the possibility that different types of histone arginine methylation are associated with different states of differentiation during cortical development, I focussed on the distribution of H4R3me2s (symmetric) and H4R3me2a (asymmetric) modifications within the developing cortex. In this report I shed some light on the dynamic distribution of the two histone arginine methyl marks during cortical differentiation. I highlight the observation that H4R3me2s is highly prevalent in the proliferating NSCs during their expansion phase and prior to the onset of differentiation and is thus likely to be associated with the “stem-like” cellular state of the NSCs. I also show that the H4R3me2a modification appears in post-mitotic neurons and early differentiating OLPs along with the H4R3me2s. I propose that this sequential activation of the different global epigenetic marks during neural development specifies the transition from the “stem-like” state of the NSCs initially, marked by H4R3me2s prevalence, to the commitment to differentiation during neurogenesis and later gliogenesis, marked by the presence of both H4R3me2s and H4R3me2a modifications.

## Results

### Distribution of symmetric and asymmetric dimethyl histone H4 modifications in the early neuroepithelium

**Figure 1:**
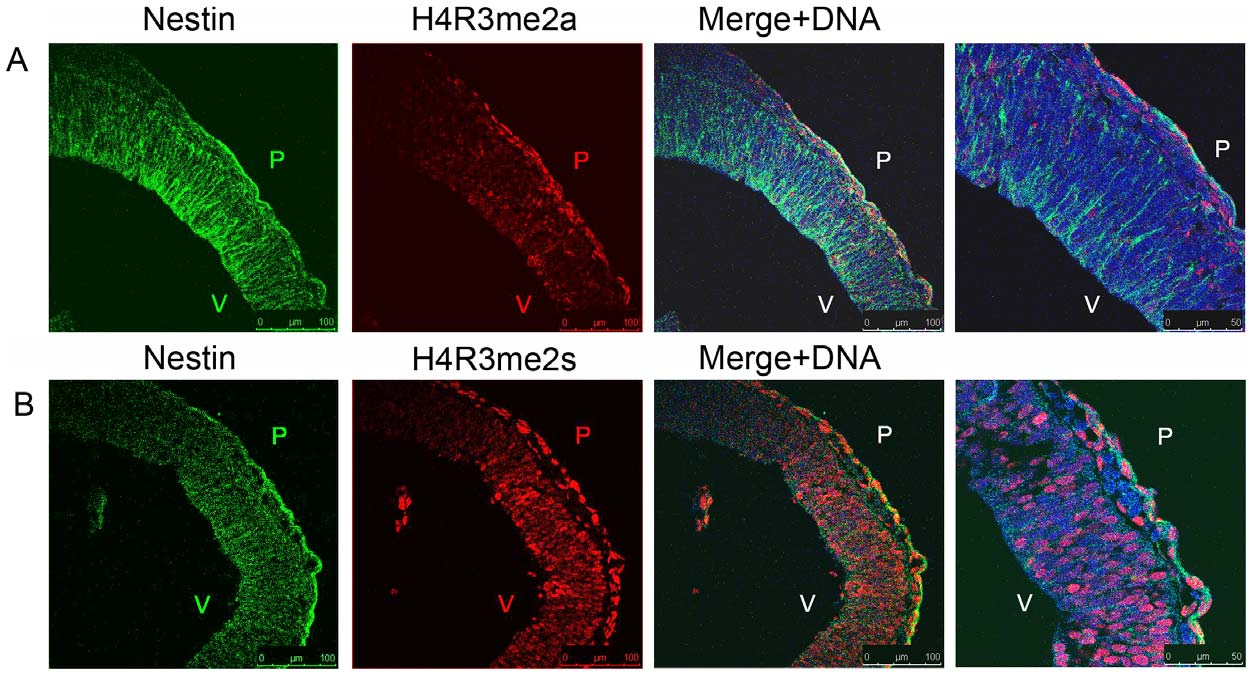
H4R3me2s, but not H4R3me2a are found in the early neural precursors at E10.5. A) Cortical neuroepithelium of E10.5 mouse embryos showing that no H4R3me2a marks are detectable in the Nestin-expressing precursor cells. B) Cortical neuroepithelium of E10.5 mouse embryos showing high levels of H4R3me2s in the Nestin-expressing precursor cells. The three left-most images are 10 magnifications, and the right-most image is a 40× magnification of the same tissue. Scale bars are indicated on the panels. P – pial, V- ventricular surfaces of the cortex.

Previous investigations of the distribution of H4R3me marks during early murine development indicated that this modification is highly dynamic at the early developmental stages and is affected by egg cytoplasmic factors [6]. However, it was not clear whether the histone arginine modifications investigated were symmetric or asymmetric. Thus, I sought to establish the distribution of H4R3me2s and H4R3me2a modifications in the developing murine cortex during the early stage of cortical development in order to determine whether they may constitute a part of the histone code specifying the different stages of neural precursor differentiation. Mouse embryos were isolated at day 10.5 of gestation (E10.5). At this stage the neuroepithelium consists mainly of a proliferating undifferentiated population of neural precursor cells [14]. Neurogenesis starts at around E12 in the murine cortex and is accompanied by a series of asymmetric divisions of neural precursor cells generating a post-mitotic neuron and another neural precursor [2]. In order to determine the distribution of H4R3me2s and H4R3me2a modifications within the developing neuroepithelium I performed immunostaining with histone modification-specific antibodies and an anti-Nestin antibody to detect undifferentiated neural precursors. At this stage most of the cells of the neuroepithelium exhibit Nestin immunoreactivity and make up the largest fraction of the developing cortex, thus allowing the identification of uncommitted neural precursors. An abundance of H4R3me2s was detected at E10.5 across the whole width of the neuroepithelium within the Nestin+ precursor cells (Fig. 1B). The neuroepithelial cells undergo the so-called interkinetic nuclear migration whereby the nuclei of these cells migrate up and down the apical-basal axis during the cell cycle [15]. During G1 the nucleus migrates from apical to basal surface, remaining at the basal side during S phase and migrating back to the apical surface during G2 phase with mitosis occurring at the apical (ventricular) surface [15]. While not all neuroepithelial cells exhibit H4R3me2s immunoreactivity, its distribution across the whole width of the epithelium makes it difficult to definitively state that the presence of H4R3me2s is regulated in a cell cycle stage-specific manner. Rather, this particular histone modification seems to be associated with the proliferative, undifferentiated cellular state of the neural precursor cells. Intriguingly, almost no H4R3me2a was detected at this stage (Fig. 1A) suggesting that this modification is not part of the proliferative programme of the neural precursors.

### Distribution of H4R3me2s and H4R3me2a in the cortex after the onset of neurogenesis

**Figure 2:**
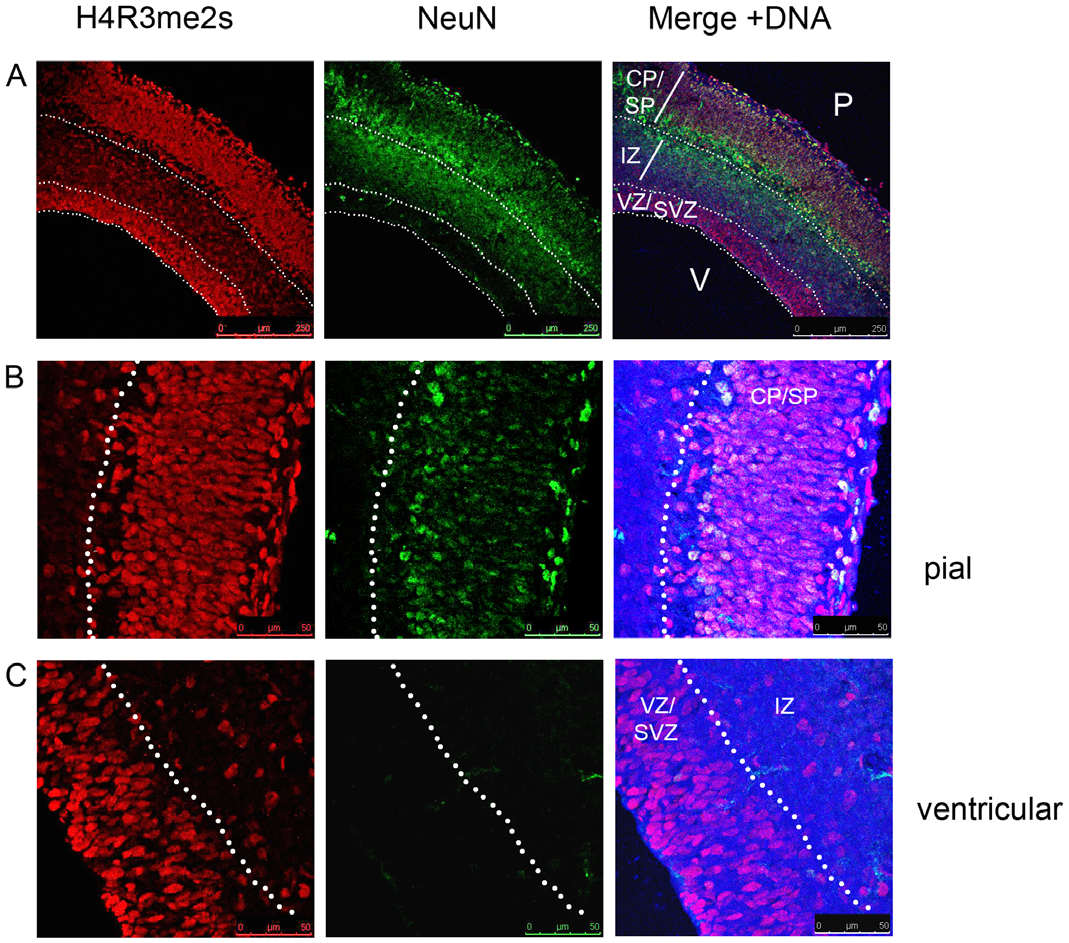
H4R3me2s is found in the neurons and in the ventricular zone of the cortex at E15.5. A) An overview of the distribution of H4R3me2s modification in the cortex at E15.5 showing a high degree of co-localisation with NeuN-expressing postmitotic neurons. B) and C) Higher magnifications of the same sections as in A) at the pial (B) and ventricular (C) surfaces showing the co-localisation of H4R3me2s with the post-mitotic neuronal marker NeuN (B) and in the neural precursors found at the ventricular zone at this stage (C). Image in panel A is 10× and in panels B and C 40× magnifications. Relevant scale bars are indicated. Abbreviation are as follows: CP – cortical plate, IZ – intermediate zone, SP – subplate, SVZ – subventricular zone, VZ – ventricular zone. P – pial, V- ventricular surfaces.

To further characterise the distribution of the H4R3me2s and H4R3me2a modifications during neural development, I stained cortices from E15.5 with the antibodies against these histone modifications and an anti-NeuN antibody to identify post-mitotic neurons. At E15.5 a very clear layering of the cerebral cortex is visible, containing ventricular and a newly formed subventricular zones, as well as the subplate and the cortical plate populated by post-mitotic neurons [16]. Staining of the telencephalon at E15.5 with an anti-H4R3me2s antibody revealed an interesting pattern of distribution. The ventricular and subventricular zones showed very high levels of this histone modification (Fig. 2A and C) as expected from the observations of proliferating neural precursors at E10.5. However, in addition to the prevalence of H4R3me2s in the ventricular zone, high levels of this modification were detected in the post-mitotic neurons expressing NeuN marker within the cortical plate and subplate (Fig. 2A and B). Intermediate zone was practically devoid of nuclei marked by the expression of H4R3me2s. Therefore, the symmetric dimethylation of histone H4 on arginine 3 is not confined to proliferating neuroepithelial cells, but is also present in the post-mitotic projection neurons of the cortex after the onset of neurogenesis.

**Figure 3:**
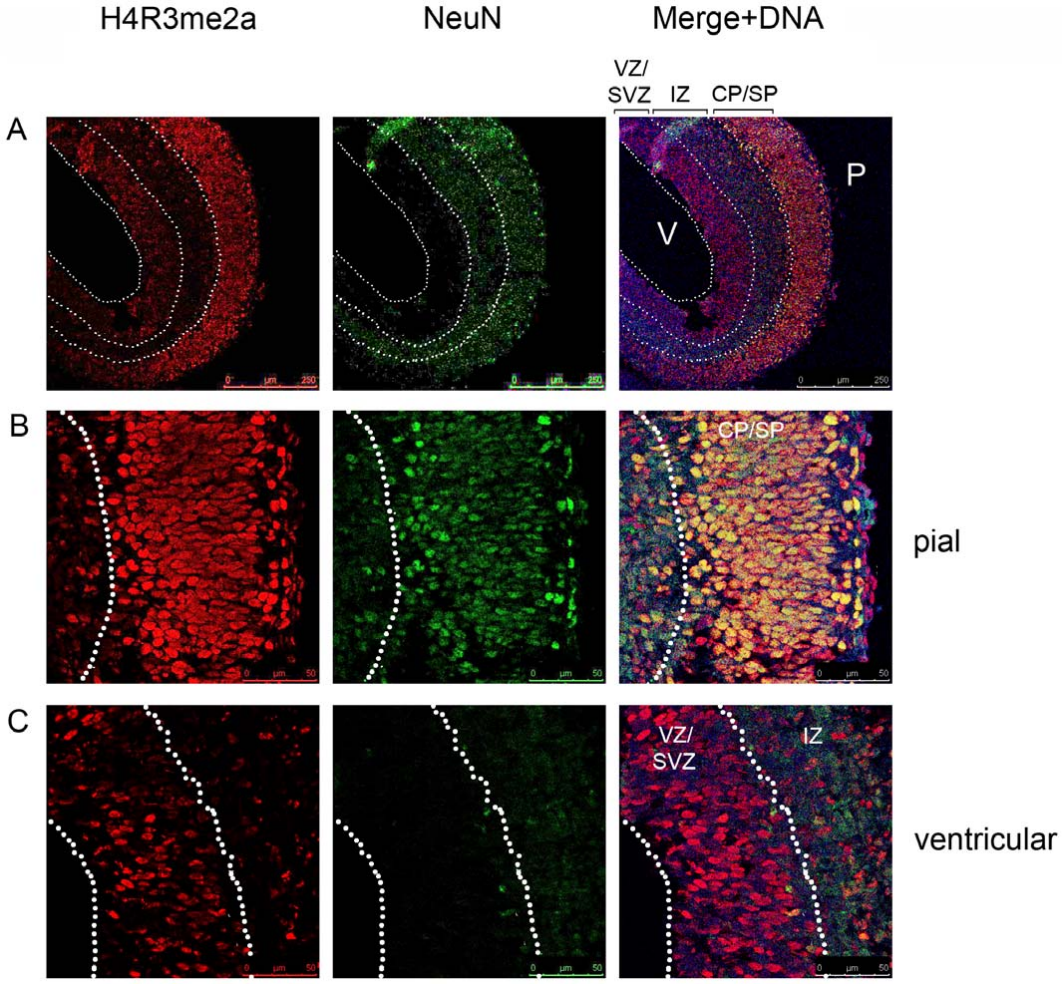
H4R3me2a is found in the neurons and in the ventricular zone of the cortex at E15.5. A) An overview of the distribution of H4R3me2a modification in the cortex at E15.5 showing a high degree of co-localisation with NeuN-expressing postmitotic neurons. B) and C) Higher magnifications of the same sections as in A) at the pial (B) and ventricular (C) surfaces showing the co-localisation of H4R3me2a with the post-mitotic neuronal marker NeuN (B) and in the neural precursors found at the ventricular zone at this stage (C). Image in panel A is 10× and in panels B and C 40× magnifications; the relevant scale bars are indicated. Abbreviation are as follows: CP – cortical plate, IZ – intermediate zone, SP – subplate, SVZ – subventricular zone, VZ – ventricular zone. P – pial, V- ventricular surfaces.

Investigation of the distribution of the H4R3me2a in the cortex at E15.5 revealed that it is found in the post-mitotic neurons of the cortical plate and the subplate and weakly in the ventricular/subventricular zones (Fig. 3A and B). The pattern of distribution was similar to the one observed with H4R3me2s. Thus, during the neurogenic phase a combination of these two modifications defines the epigenetic state of post-mitotic neurons, but interestingly also the precursor cells found in the ventricular zone, suggesting that the later stage neural precursors are somewhat different from their early counterparts in their epigenetic profile.

**Figure 4:**
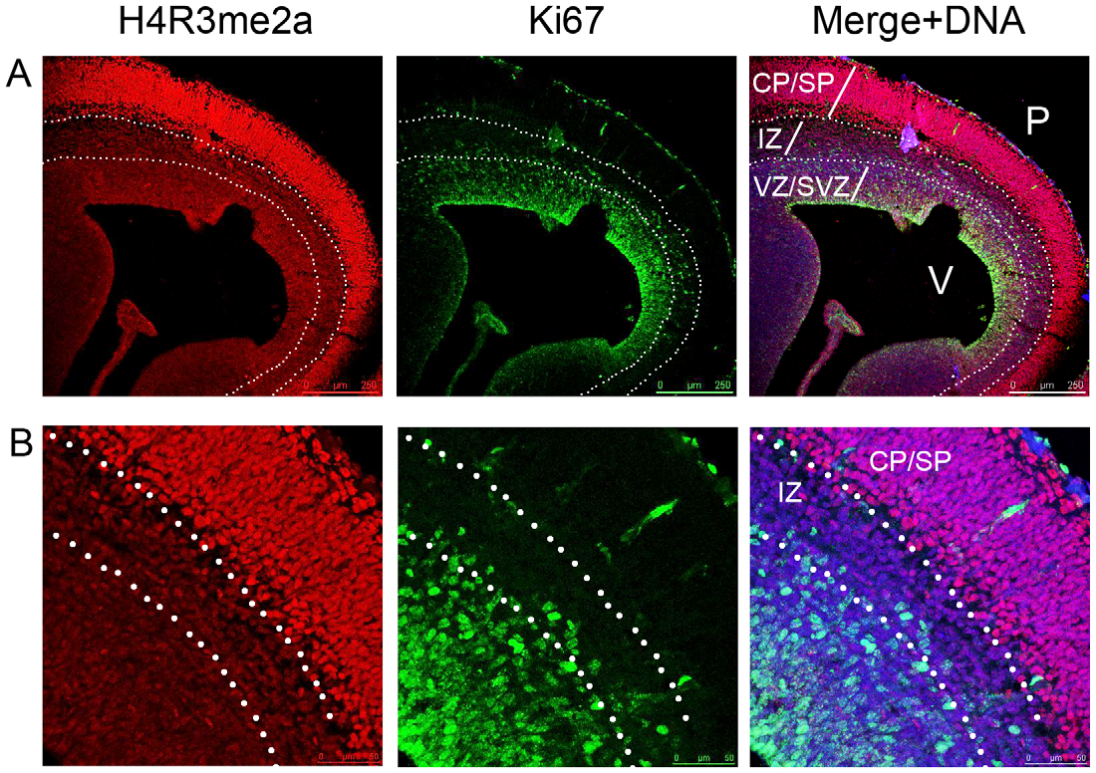
H4R3me2a does not co-localise with proliferating neural precursors of the ventricular zone. A) An overview of H4R3me2a modifications in the cortex at E15.5 showing no co-staining with the proliferating cell marker Ki67. B) Higher magnification of the same section showing mutually exclusive localisations of Ki67-expressing proliferating precursors and H4R3me2a. Image in panel A is 10× and in panel B 40× magnifications. The relevant scale bars are indicated. Abbreviation are as follows: CP – cortical plate, IZ – intermediate zone, SP – subplate, SVZ – subventricular zone, VZ – ventricular zone. P – pial, V – ventricular surfaces.

In order to further characterise the association of H4R3me2a with the proliferative or post-mitotic state of the neural cells, cortices isolated from E15.5 embryos were co-immunolabelled with antibodies against H4R3me2a and Ki67, a marker of proliferating cells. As shown in Fig. 4A and B, the post-mitotic neurons of the cortical plate and subplate show very high levels of H4R3me2a, with very low levels being detected in the ventricular and the subventricular zones. Ki67 is found primarily in the ventricular and subventricular zones of the cortex (Fig. 4A and B). The distribution of H4R3me2a and Ki67 is virtually mutually exclusive, although some cell can be detected which are marked by low levels of Ki67 and H4R3me2a immunoreactivity. Thus, H4R3me2a is mainly associated with post-mitotic neurons and only weakly with neural precursors, possibly marking those precursor cells which are ready to commit to differentiation.

### Distribution of H4R3me2s and H4R3me2a modifications in oligodendrocyte precursors

NSCs give rise to glial cells at a later stage of embryonic cortical development and continuing into post-natal period [4]. To investigate whether the two histone H4 arginine modifications are also present in the developing oligodendrocyte progenitors (OLPs) I performed immunolabelling of murine cortices from E15.5. At this stage the early committed OLPs can be detected by staining with an anti-PDGFR-α antibody [17], [18], [19], [20]. The OLPs are initially specified as proliferative, migratory precursor cells and do not become post-mitotic shortly after their specification as the neurons do [17]. The initial appearance of the OLPs is distinctly radial and many of these cells co-label with a radial glial marker RC2 [21], suggesting that these cells might form by direct transformation of radial glia [21], [22], [23]. Therefore, OLPs at their initial stages may represent a highly plastic population of cells still capable of transforming into other cellular types. In this respect it is of interest to understand the epigenetic regulation of their re-programming during development of the cortex.

**Figure 5:**
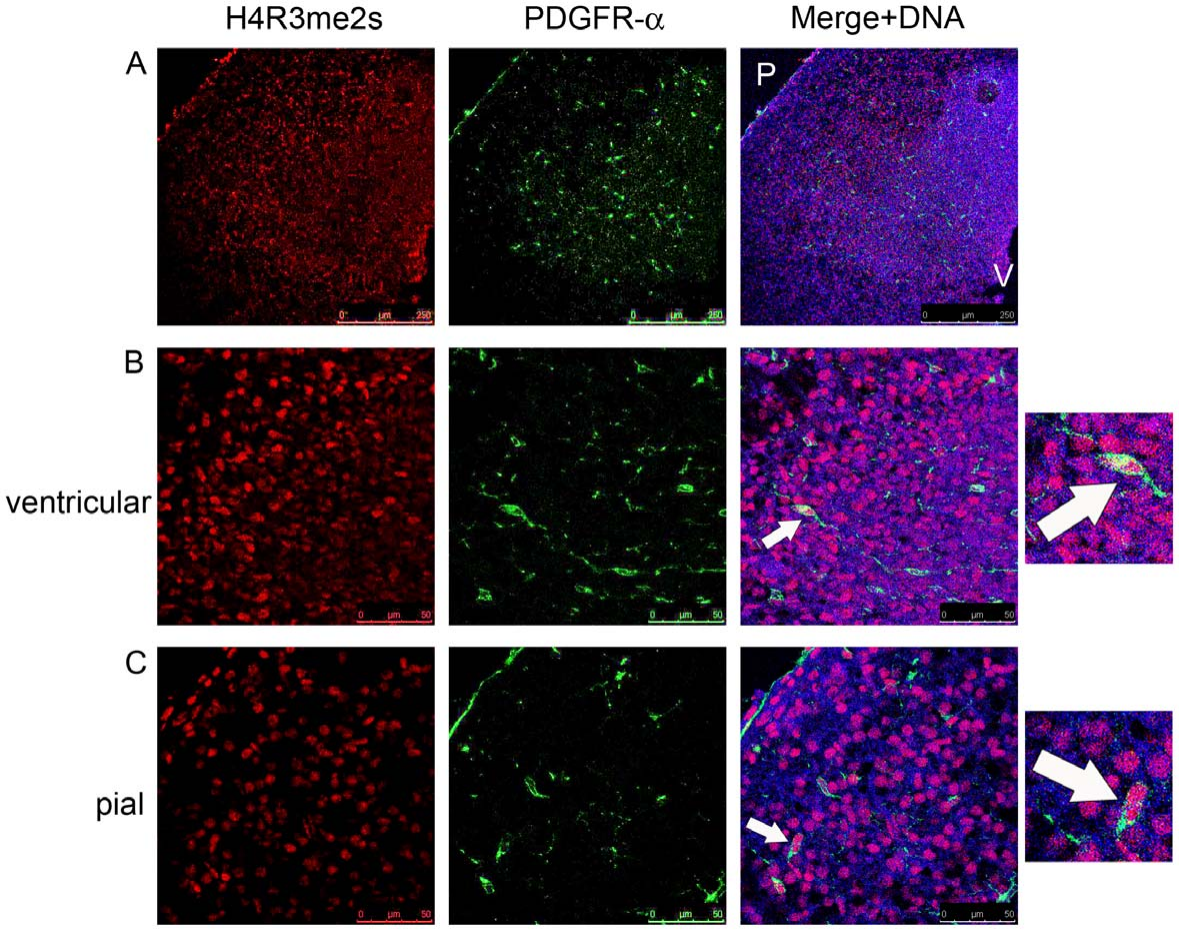
H4R3me2s modification is found in some OLPs expressing PDGFR-α. A) An overview of H4R3me2s modifications in the cortex at E15.5 showing some co-localisation with PDGFR-α expressing OLPs. B) and C) Higher magnification of the same sections showing H4R3me2s positive PDGFR-α OLPs present near the ventricular (B) and pial (C) surfaces of the cortex at E15.5. To the right are magnified images of the highlighted OLP cells showing H4R3me2s immunoreactivity. Image in panel A is 10× and in panel B 40× magnifications. The relevant scale bars are indicated. P – pial, V – ventricular surfaces. Arrows indicate OLPs which are marked by the presence of H4R3me2s modifications.

PDFGR-α expressing OLPs are scattered throughout the cortex at E15.5. The staining of cortices isolated at E15.5 with antibodies against H4R3me2s modifications revealed that PDGFR-α expressing OLPs are heterogeneous with respect to the presence of this histone modification (Fig. 5A–C). Some cells contain this modification, but others do not. It is not clear whether this heterogeneity reflects OLPs at different stages of their differentiation, or whether different subtypes of OLPs are marked by the difference in this histone modification [17].

**Figure 6:**
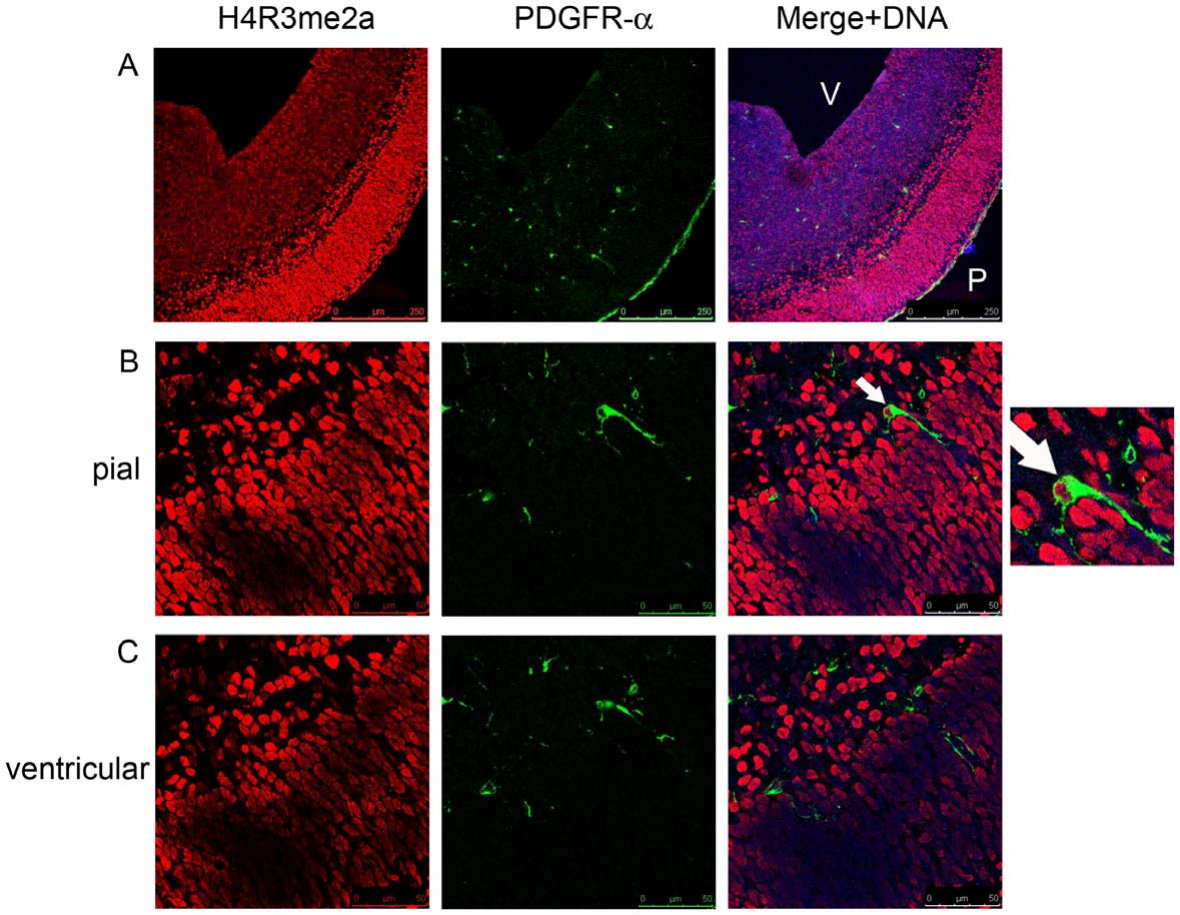
H4R3me2a modification is found in some OLPs expressing PDGFR-α. A) An overview of H4R3me2a modifications in the cortex at E15.5 showing some co-localisation with PDGFR-α expressing OLPs. B) and C) Higher magnification of the same sections showing H4R3me2a positive PDGFR-α OLPs present near the pial (B) and ventricular (C) surfaces of the cortex at E15.5. To the right of panel (B) is a magnified image of the highlighted OLP cell showing H4R3me2s immunoreactivity. Image in panel A is 10× and in panel B 40× magnifications. The relevant scale bars are indicated. P – pial, V – ventricular surfaces. Arrows indicate OLPs which are marked by H4R3me2a modifications.

Staining of PDGFR-α-expressing OLPs with an antibody against H4R3me2a modification revealed that the OLPs are also heterogeneous with respect to this histone modification, some staining positive and others negative for its presence (Fig. 6A–C). As is the case with H4R3me2s, it is not clear whether the different distribution of the H4R3me2a modification represents different stages of differentiation and commitment of the OLPs or just marks a distinct subclass of these precursors. In this respect, OLPs appear to be different from both the early neural precursors (at E10.5) and the post-mitotic projection neurons detected at E15.5 in the cortex. The early precursors appear to be specified by the presence of H4R3me2s modification and the absence of H4R3me2a modification. The projection neurons of the cortex are marked by the presence of both, symmetric and asymmetric H4R3me2 modifications. The OLPs represent yet another class of precursor cells which are heterogeneous with respect to the presence of H4R3me2s and H4R3me2a. However, we cannot at the present time determine whether both modifications can be found within the same OLP cell as there are no antibodies which would allow co-staining of these cells with both of them simultaneously.

## Discussion

This study was carried out to test the possibility that two distinct post-translation modifications of histone H4 on arginine 3 residue undergo reprogramming during cortical development and differentiation. The results of the study support the notion that sequential activation of these two modifications specifies the transition from the “stem-like” neural precursor state to a more differentiated cellular state. Thus, the symmetric H4R3me2 is associated with the undifferentiated neural precursors, whereas the combination of H4R3me2s and H4R3me2a forms a signature of the post-mitotic neurons and some differentiating OLPs.

Post-translation histone modifications and the various combinations thereof have been proposed to “write” the “histone code” creating extensive diversity through such combinatorial sets of modifications for the recruitment of distinct regulatory proteins and complexes [24]. As such, particular sets of histone modifications can actually represent specific epigenetic signatures of differentiating cells during development. In this report, I have focussed on the distribution of two distinct and very poorly characterised histone modifications, H4R3me2s and H4R3me2a, during cortical development and differentiation. Arginine methylation is a prevalent post-translation modification which regulates many physiological processes. Importantly, there is evidence that histone arginine methylation or at least specific types of it is associated with differentiation [10], [11], [25]. Intriguingly, all of these examples involve the PRMTs of type I subclass which mediate asymmetrical dimethylation of arginines. On the other hand, type II arginine methyltransferase, PRMT5, has been implicated in the repression of genes leading to differentiation of different cell types at early stages of development. In primordial germ cells (PGC), PRMT5 represses a target gene necessary for PGC differentiation [12]. In primary erythroid progenitors, PRMT5 mediates repression of developmentally regulated β-globin locus [13]. The involvement of type I and type II PRMTs is not always clear cut with respect to their roles in the maintenance of “stem-like” or the more differentiated cellular state. Possibly, in some cell types the sequential activation of the two different PRMT subclasses may specify the progressive restriction of multipotency and a transition from a “stem-like” to a more differentiated and developmentally restricted cellular state. Importantly, in cortical development, the type I PRMT, PRMT1, is likely to be involved in the commitment of the neural precursors to become a post-mitotic neuron since a protein which stimulates the activity of PRMT1, BTG2/TIS21, is expressed exclusively in neural precursor cells which are committed to become post-mitotic neurons [25]. Furthermore, there is evidence that the nerve growth factor (NGF)-induced differentiation of PC12 cells increases the amount of total arginine methylated proteins and that this process is mediated by PRMT1 as well [26], [27].

It is interesting that both the major PRMTs of type I and type II, i.e., PRMT1 and PRMT5, respectively, can methylate the same residue on histone H4, but leading to opposing transcriptional outcomes [9]. Whilst it is not clear whether both enzymes are active at the same regulatory regions of genes, it is intriguing to speculate that levels of gene transcription can be “fine-tuned” by the combined actions of PRMT1 and 5, for example at the same regulatory sequences. Such fine-tuning of gene transcription might be instrumental in directing the differentiation events during cortical development. It will be very important to identify the signalling pathways which regulate the activity of the PRMTs which are responsible for H4R3me2a and H4R3me2s deposition during cortical development as well as find out exactly which PRMTs are responsible for these modifications and how these enzymes are targeted to the specific gene regulatory elements [28]. Furthermore, it will be important to identify the “transcriptomes” regulated by these modifications in the developing cortex and more specifically in the different neural cell lineages as they progress through their developmental programmes.

## Materials and Methods

### Ethics statement

All animal experiments were approved by the University College London local ethical committee and conformed to the UK Animals (Scientific Procedures) Act 1986. Project license number PPL 70/6697.

### Embryo Immunofluorescence

E10.5 and E15.5 embryos were collected from CD1 mice, rinsed in PBS and fixed in 4% paraformaldehyde (PFA) at 4°C for 1–2 hours. Embryos were then cryoprotected in 30% sucrose-PBS and subsequently mounted in O.C.T. (Tissue-Tek) on dry ice. Mounted embryos were sectioned at 10 µm using a Leica cryostat and used for immunofluorescence after air-drying for at least 1 hour. Sections with the tissue were permeabilised with cold 100% methanol for 3 minutes at −20°C, rinsed three times in PBS and blocked for 1 hour at room temperature (RT) using the following blocking solution: PBS/0.1% TritonX-100, 10% normal goat serum. Primary antibodies were added in the blocking solution overnight at 4°C. Sections were washed three times in PBS at RT and incubated with fluorescently labelled secondary antibodies and Hoechst for 1 hour at RT. Subsequently, the sections were rinsed in PBS three times and quickly in water and mounted using DAKO mounting medium.

### Antibodies Used

I used the following primary antibodies in this study: anti-H4R3me2s (Abcam, rabbit polyclonal, 1∶1000), anti-H4R3me2a (Active Motif, rabbit polyclonal, 1∶1000), anti-NeuN (Chemicon Temecular, CA, mouse monoclonal, 1∶500), anti-PDGFRα (BD Sciences, rat polycolonal, 1∶400), anti-Nestin (Santa Cruz, mouse monoclonal, 1∶400). The following secondary antibodies were used: goat anti-mouse Alexa 488 (1∶1000), goat anti-rabbit Alexa 568 (1∶1000), goat anti-rat Alexa 488 (1∶500). All secondary antibodies were purchased from Invitrogen. Fluorescent images were taken with a Leica Microsystems CMS confocal microscope using either 10× or 40× (oil) objective lenses. For each panel, attenuation, contrast, brightness and pinhole aperture remained constant. Approximately 10 optical sections were analysed per section with only one optical section being shown. Therefore, only the nuclei within the section are shown. Embryos were collected from different pregnant mice and the results presented were highly reproducible.

## References

[1] Hirabayashi Y, Gotoh Y (2010). Epigenetic control of neural precursor cell fate during development.. Nature Reviews Neuroscience.

[2] Okano H, Temple S (2009). Cell types to order: temporal specification of CNS stem cells.. Current Opinion in Neurobiology.

[3] Guillemot F (2007). Cell fate specification in the mammalian telencephalon.. Progress in Neurobiology.

[4] Miller FD, Gauthier AS (2007). Timing is everything: Making neurons versus glia in the developing cortex.. Neuron.

[5] Hsieh J, Gage FH (2004). Epigenetic control of neural stem cell fate.. Current Opinion in Genetics & Development.

[6] Sarmento OF, Digilio LC, Wang YM, Perlin J, Herr JC (2004). Dynamic alterations of specific histone modifications during early murine development.. Journal of Cell Science.

[7] Zhang Y, Reinberg D (2001). Transcription regulation by histone methylation: interplay between different covalent modifications of the core histone tails.. Genes & Development.

[8] Jenuwein T, Allis CD (2001). Translating the histone code.. Science.

[9] Bedford MT, Clarke SG (2009). Protein Arginine Methylation in Mammals: Who, What, and Why.. Molecular Cell.

[10] Chen SL, Loffler KA, Chen DG, Stallcup MR, Muscat GEO (2002). The coactivator-associated arginine methyltransferase is necessary for muscle differentiation - CARM1 coactivates myocyte enhancer factor-2.. Journal of Biological Chemistry.

[11] Berthet C, Guehenneux F, Revol V, Samarut C, Lukaszewicz A (2002). Interaction of PRMT1 with BTG/TOB proteins in cell signalling: molecular analysis and functional aspects.. Genes to Cells.

[12] Ancelin K, Lange UC, Hajkova P, Schneider R, Bannister AJ (2006). Blimp1 associates with Prmt5 and directs histone arginine methylation in mouse germ cells.. Nature Cell Biology.

[13] Zhao Q, Rank G, Tan YT, Li HT, Moritz RL (2009). PRMT5-mediated methylation of histone H4R3 recruits DNMT3A, coupling histone and DNA methylation in gene silencing.. Nature Structural & Molecular Biology.

[14] Bayer SA, Altman J (1991).

[15] Gotz M, Huttner WB (2005). The cell biology of neurogenesis.. Nature Reviews Molecular Cell Biology.

[16] Dehay C, Kennedy H (2007). Cell-cycle control and cortical development.. Nature Reviews Neuroscience.

[17] Kessaris N, Pringle N, Richardson WD (2008). Specification of CNS glia from neural stem cells in the embryonic neuroepithelium.. Philosophical Transactions of the Royal Society B-Biological Sciences.

[18] Pringle NP, Richardson WD (1993). A SINGULARITY OF PDGF ALPHA-RECEPTOR EXPRESSION IN THE DORSOVENTRAL AXIS OF THE NEURAL-TUBE MAY DEFINE THE ORIGIN OF THE OLIGODENDROCYTE LINEAGE.. Development.

[19] Tekki-Kessaris N, Woodruff R, Hall AC, Gaffield W, Kimura S (2001). Hedgehog-dependent oligodendrocyte lineage specification in the telencephalon.. Development.

[20] Spassky N, Goujet-Zalc C, Parmantier E, Olivier C, Martinez S (1998). Multiple restricted origin of oligodendrocytes.. Journal of Neuroscience.

[21] Fogarty M, Richardson WD, Kessaris N (2005). A subset of oligodendrocytes generated from radial glia in the dorsal spinal cord.. Development.

[22] Choi BH, Kim RC, Lapham LW (1983). DO RADIAL GLIA GIVE RISE TO BOTH ASTROGLIAL AND OLIGODENDROGLIAL CELLS.. Developmental Brain Research.

[23] Hirano M, Goldman JE (1988). GLIOGENESIS IN RAT SPINAL-CORD - EVIDENCE FOR ORIGIN OF ASTROCYTES AND OLIGODENDROCYTES FROM RADIAL PRECURSORS.. Journal of Neuroscience Research.

[24] Fischle W, Wang YM, Allis CD (2003). Histone and chromatin cross-talk.. Current Opinion in Cell Biology.

[25] Iacopetti P, Michelini M, Stuckmann I, Oback B, Aaku-Saraste E (1999). Expression of the antiproliferative gene TIS21 at the onset of neurogenesis identifies single neuroepithelial cells that switch from proliferative to neuron-generating division.. Proceedings of the National Academy of Sciences of the United States of America.

[26] Cimato TR, Tang J, Xu Y, Guarnaccia C, Herschman HR (2002). Nerve growth factor-mediated increases in protein methylation occur predominantly at type I arginine methylation sites and involve protein arginine methyltransferase 1.. Journal of Neuroscience Research.

[27] Cimato TR, Ettinger MJ, Zhou XB, Aletta JM (1997). Nerve growth factor-specific regulation of protein methylation during neuronal differentiation of PC12 cells.. Journal of Cell Biology.

[28] Chittka A, Chittka L (2010). Epigenetics of Royalty.. PLoS Biol.

